# Correction: Sainz et al. Analysis of Thioredoxins and Glutaredoxins in Soybean: Evidence of Translational Regulation under Water Restriction. *Antioxidants* 2022, *11*, 1622

**DOI:** 10.3390/antiox12071377

**Published:** 2023-07-03

**Authors:** María Martha Sainz, Carla Valeria Filippi, Guillermo Eastman, José Sotelo-Silveira, Omar Borsani, Mariana Sotelo-Silveira

**Affiliations:** 1 Laboratorio de Bioquímica, Departamento de Biología Vegetal, Facultad de Agronomía, Universidad de la República, Avenida Garzón 780, Montevideo 12900, Uruguay; 2 Departamento de Genómica, Instituto de Investigaciones Biológicas Clemente Estable, MEC, Av. Italia 3318, Montevideo 11600, Uruguay; 3 Department of Biology, University of Virginia, 485 McCormick Rd., Charlottesville, VA 22904, USA; 4 Departamento de Biología Celular y Molecular, Facultad de Ciencias, Universidad de la República, Iguá 4225, Montevideo 11400, Uruguay

In the original publication [1], there was a mistake in Figure 4 as published. The scale of Figure 4 was wrong. The corrected Figure 4 appears below. The authors state that the scientific conclusions are unaffected. This correction was approved by the Academic Editor. The original publication has also been updated.

## Figures and Tables

**Figure 4:**
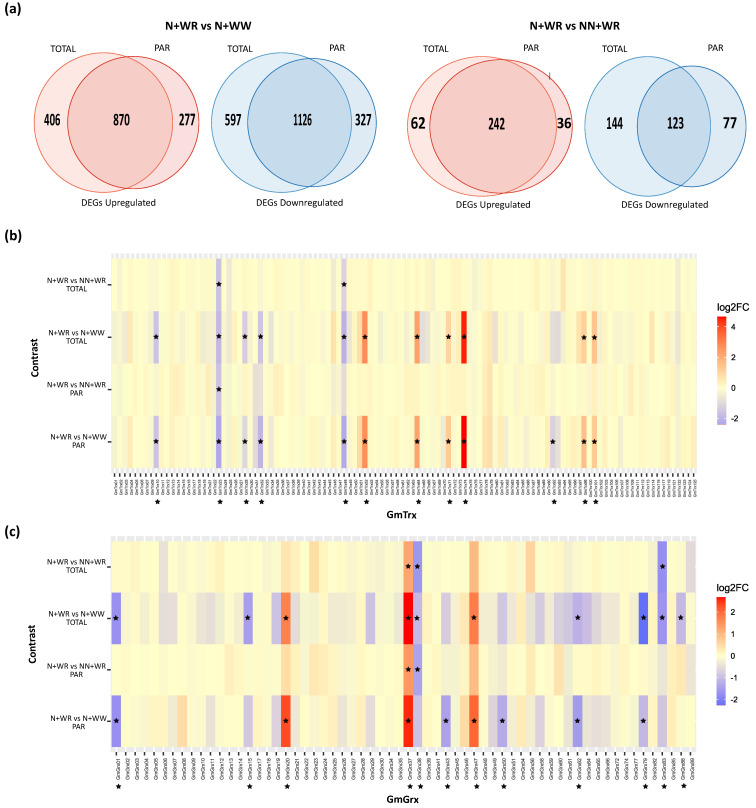
Differentially expressed gene (DEG) analysis and *GmTrx* and *GmGrx* expression profiles in nodulated (N) and water-restricted (WR) plants with respect to well-watered (WW) and non-nodulated (NN) plants. (**a**) Venn diagrams showing up- and down-regulated genes in the N+WR vs. N+WW and N+WR vs. NN+WR contrasts in total RNA (TOTAL) and polysome-associated mRNA (PAR) fractions. (**b**) Expression profiles of *GmTrx*. (**c**) Expression profiles of *GmGrx*. Heatmaps were constructed from the RNA-seq experimental data. Asterisks indicate the differentially expressed *GmTrx* and *GmGrx* genes found in our study. Genes with |log2FC| > 1 and adjusted *p*-value (padj) < 0.05 were considered differentially expressed.
