# Supplementary material for: Analysis of Thioredoxins and Glutaredoxins in Soybean: Evidence of Translational Regulation under Water Restriction
Source: Antioxidants (Basel). 2022 Aug 21;11(8):1622. doi: 10.3390/antiox11081622 (PMC9405309; doi:10.3390/antiox11081622)
Supplement: Supplementary file 1 [file antioxidants-11-01622-s001.zip › antioxidants-1849242-supplementary.pdf]

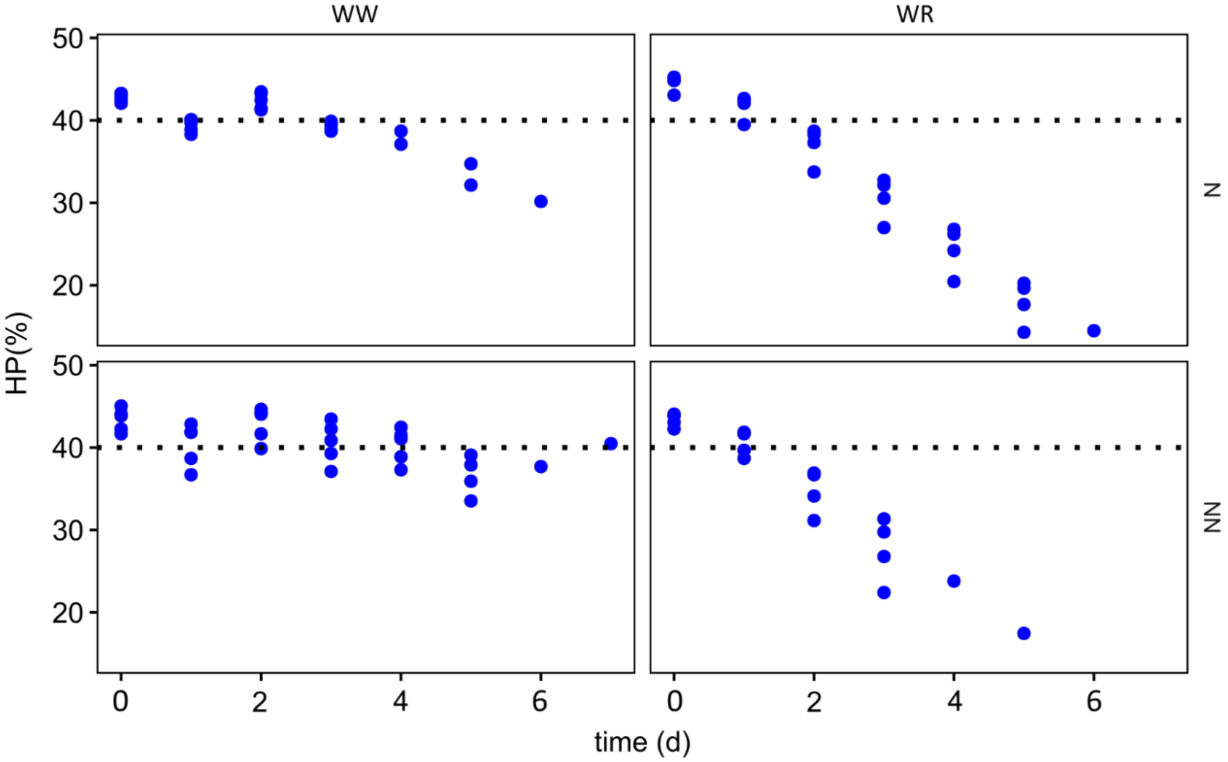

Figure S1. Substrate water retention curves from plants comprising the four combined treatments during the water deficit period (5 to 7 days). WW: well-watered plants; WR: water-restricted plants; N: nodulated plants; NN: non-nodulated plants. HP (%): amount of water in the substrate (sand:vermiculite, 1:1) expressed as a % of its dry weight. The dotted line indicates the HP (%) at which the substrate is at its field capacity (40% in the case of the substrate used in this assay).

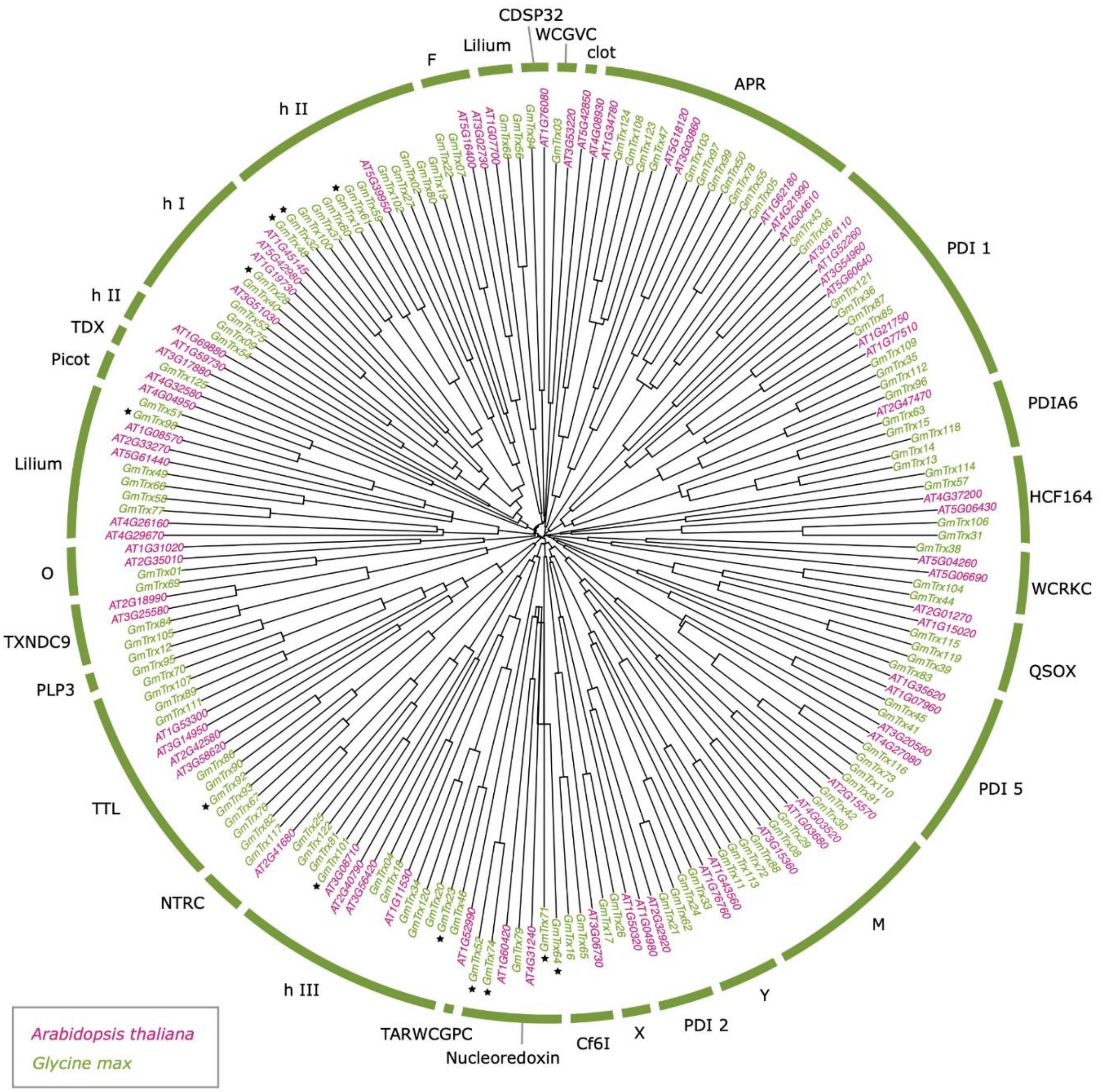

Figure S2. Thioredoxin Gene Family of *Glycine max*. *G. max* and *A. thaliana* amino acid sequences were aligned using ClustalW, as implemented in the R package msa [29]. The phylogenetic relationship of thioredoxins (Trxs) shown in the unrooted tree was estimated using multiple sequence alignment as input in phangorn [30] to construct the neighbor-joining phylogenetic trees. Active site signatures are indicated with a line outside the tree.

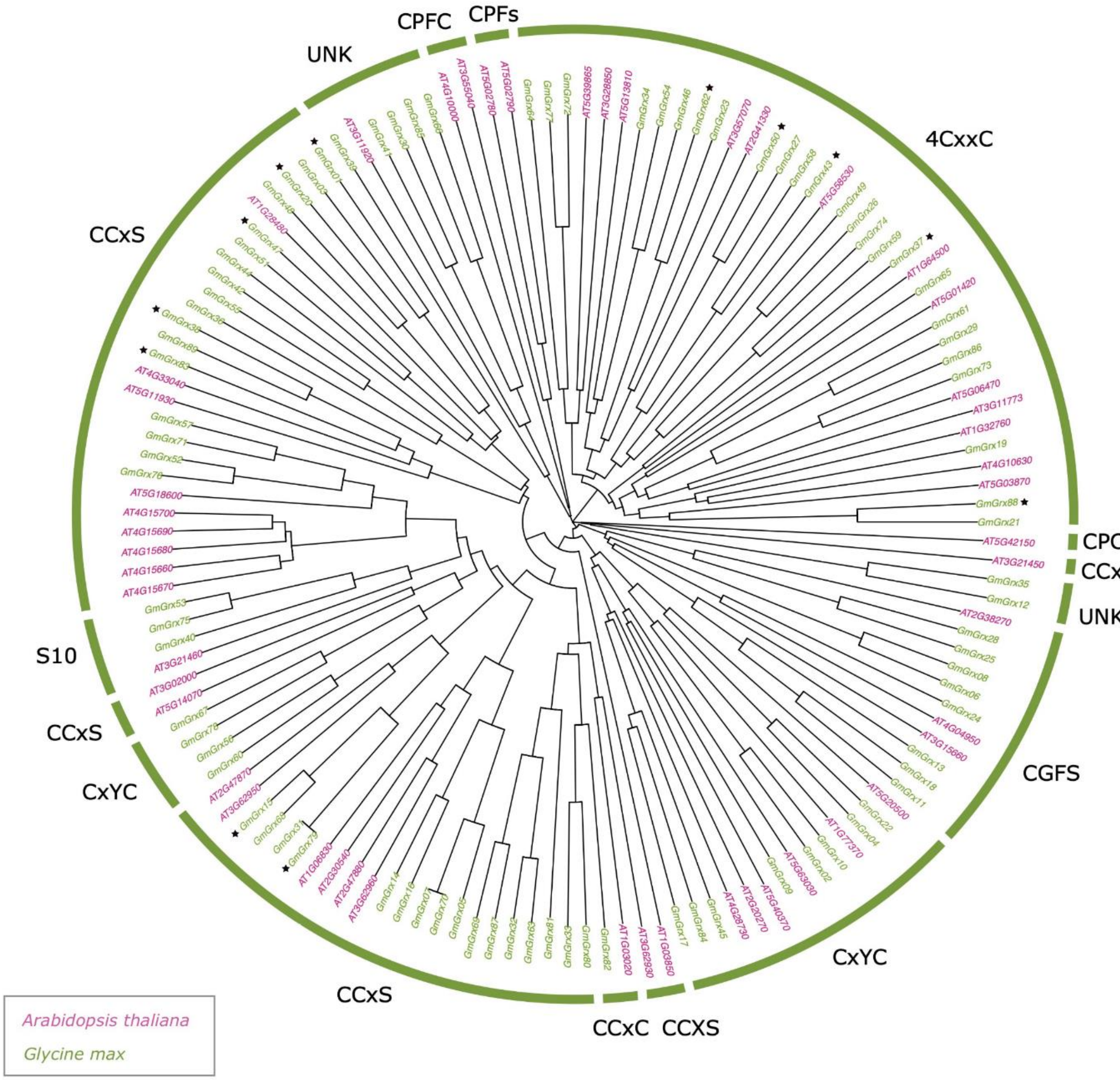

Figure S3. Glutaredoxin Gene Family of *Glycine max*. *G. max* and *A. thaliana* amino acid sequences were aligned using ClustalW, as implemented in the R package msa [29]. The phylogenetic relationship of glutaredoxins (Grxs) shown in the unrooted tree was estimated using multiple sequence alignment as input in phangorn [30] to construct the neighbor-joining phylogenetic trees. Active site signatures are indicated with a line outside the tree.

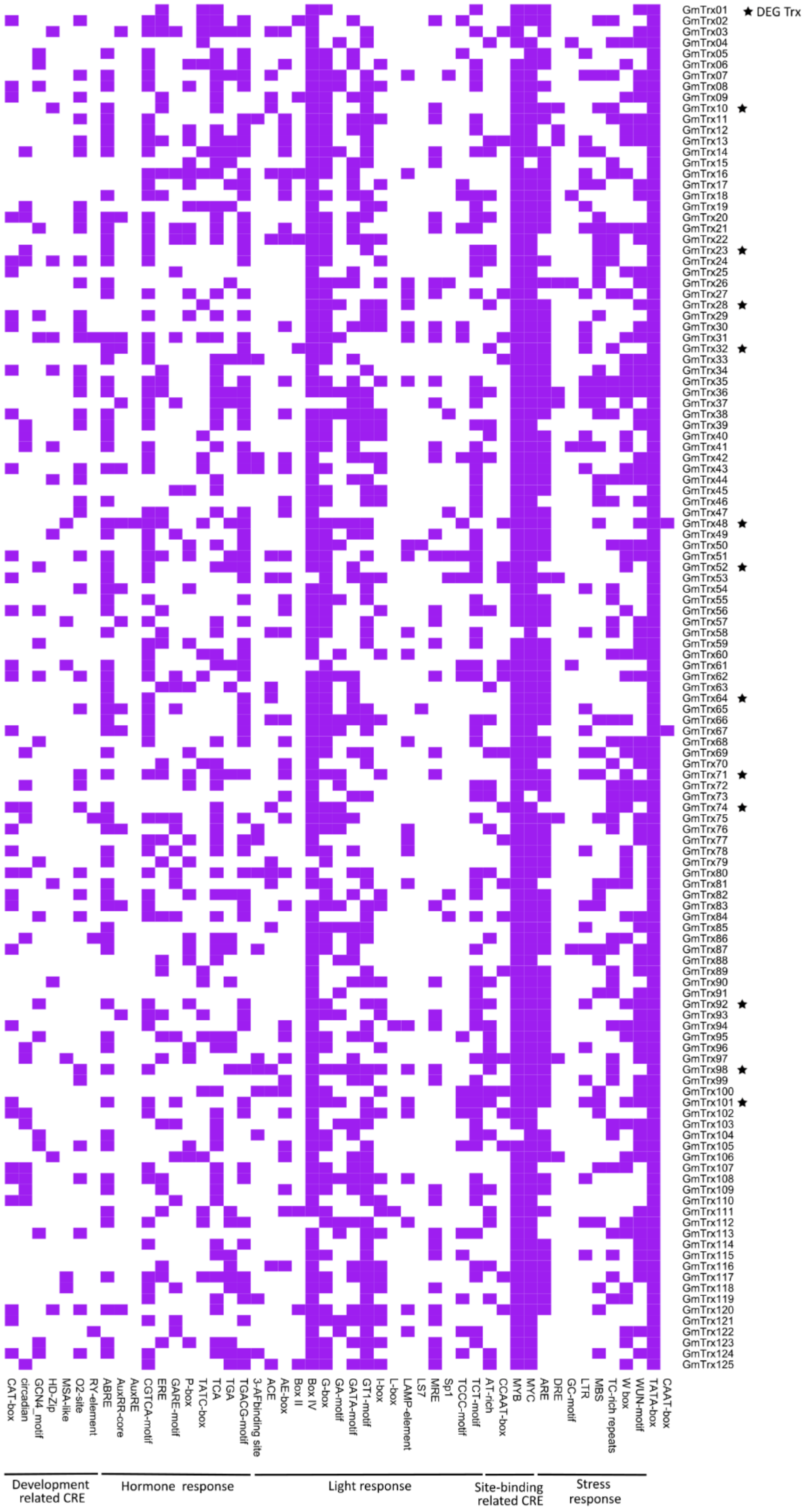

Figure S4. Prediction of cis-acting elements in the promoter sequences of *Glycine max thioredoxins (GmTrx)*. The 2,000 bp sequences upstream of each GmTrx gene were analyzed with PlantCARE [24].

Distribution of Development related CRE, hormone response, Light response, Site-binding related CRE and Stress response in *GmTrxs*.

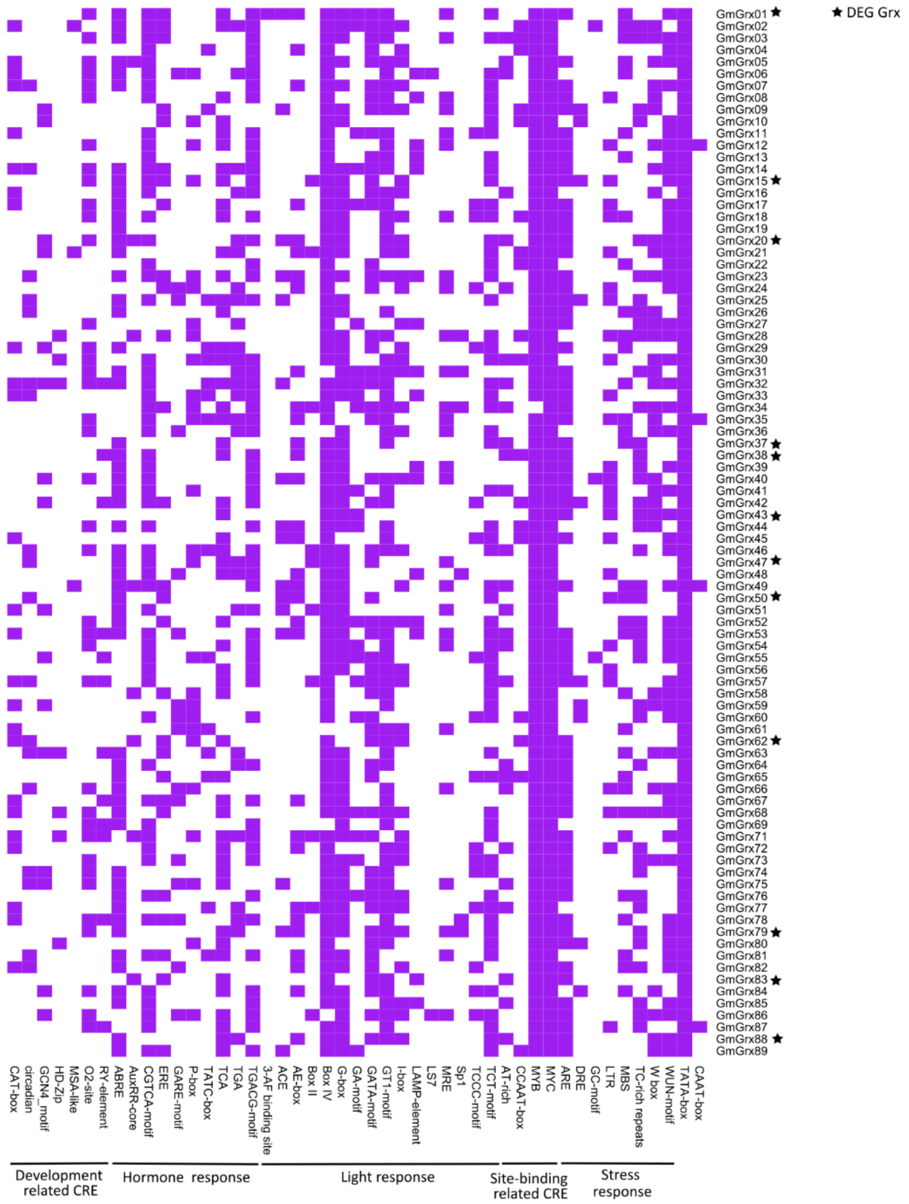

Figure S5. Prediction of cis-acting elements in the promoter sequences of *Glycine max glutaredoxins (GmGrxs)*. The 2,000 bp sequences upstream of each GmGrx gene were analyzed with PlantCARE [24]. Distribution of Development related CRE, hormone response, Light response, Site-binding related CRE and Stress response in *GmGrxs*.
